# The Response of Iranian Melon (*Cucumis melo* L.) Accessions to 2,4-D Drift

**DOI:** 10.3390/plants10112442

**Published:** 2021-11-12

**Authors:** Rouzbeh Zangoueinejad, Behnaz Sirooeinejad, Mohammad Taghi Alebrahim, Ali Ahsan Bajwa

**Affiliations:** 1 Department of Plant Production and Genetic, Faculty of Agricultural Sciences & Natural Resources, University of Mohaghegh Ardabili, Ardabil 56199-11367, Iran; r.zangoueinejad@uma.ac.ir (R.Z.); m_ebrahim@uma.ac.ir (M.T.A.); 2 Department of Horticultural Sciences, University of Tehran, Karaj 14179-35840, Iran; bz_sirooee@ut.ac.ir; 3 Weed Research Unit, New South Wales Department of Primary Industries, Wagga Wagga, NSW 2650, Australia

**Keywords:** herbicide drift, melon production, horticultural crop, herbicide damage, off-target application

## Abstract

One of the most widely used auxinic herbicides in southern Iran’s cereal crop fields is 2,4-D; however, the concurrent growing season of off-season melons in this region potentially leads to herbicide drift from cereal fields to the melon fields. To study the response of some Iranian wild melon accessions to three simulated drift rates of 2,4-D, including 112.1, 11.2, and 3.7 g ae ha^−1^, a field experiment was conducted during 2019 and 2020 growing seasons. It was found that by increasing the herbicide rate from 3.7 to 112.1 g ae ha^−1^, the level of visual injury increased in all accessions. However, significant variation in herbicide tolerance was observed among different melon accessions. The MEL-R1 was the most tolerant accession with only 20% injury, while MEL-D8 displayed very high injury rate (*ca.* 90%) as assessed at 6 weeks after treatment during 2019. The accession MEL-S3 was the most tolerant to 2,4-D drift rates (20% injury) at 6 weeks after treatment during 2020. There was no significant difference between the accessions MEL-R1 and MEL-S3 in terms of their response to 2,4-D treatment during both years of the study, as these accessions fully recovered from injury over 6 weeks after herbicide treatment. In addition, only these two accessions were able to produce yield after the application of 2,4-D at the highest rate tested (112.1 g ae ha^−1^). Therefore, the melon accessions MEL-R1 and MEL-S3 could be recommended for cultivation and even for breeding programs in order to develop 2,4-D-tolerant commercial cultivars in regions where this herbicide is commonly used in cereal crop production adjacent to the melon fields.

## 1. Introduction

Melons (*Cucumis melo* L.) are one of the off-season crops in the southern provinces of Iran [1]. Usually, spring and summer are the common seasons to grow melons in the northern hemisphere. However, growing melons in the southern Iran during typical seasons is difficult due to harsh weather conditions prevailing from mid-spring to the end of summer. Therefore, farmers grow specialty crops such as melons from mid-winter to mid-spring in these regions. In Iran, approximately 20% of the total melon production (up to 170,000 tons) was harvested in four southern provinces, including Bushehr, Hormozgan, Sistan and Balochistan, and Khozetan in 2019 [1]. Simultaneous growing season of melons and cereals in those areas has caused significant concerns for the melon growers, as one of the main herbicide options to control the infestations of some broadleaf weeds such as common mallow (*Malva neglecta* Wallr.) and field bindweed (*Convolvulus arvensis* L.) in southern Iran’s wheat (*Triticum aestivum* L.) and barley (*Hordeum vulgare* L.) fields is 2,4-D, and its off-target drift is very common. Drift is defined as physical movement of an herbicide through air, at the time of application or soon thereafter, to any site other than that intended. Herbicides may move to nontarget areas by physical spray-particle drift, vapor drift, and herbicide-contaminated soil. According to recent estimate, 2,4-D drift from cereal crop fields to melon fields cost more than USD 1 million annually due to crop damage [1]. Crop losses due to auxin herbicide drift are well reported in various crops around the world [2,3,4,5]. For instance, Culpepper et al. (2018) reported severe crop damage and yield reductions in watermelon (*Citrullus lanatus* Thunb.) crops exposed to the off-target drift of 2,4-D or dicamba. Some other examples of the crops that experience damage and visual injury due to drift rates (often very low rates compared to the label rates) of auxinic herbicides include cotton (*Gossypium hirsutum* L.) [6,7], soybean (*Glycine max* L.) [8,9], tomato (*Solanum lycopersicum* L.) [10,11], grape (*Vitis vinifera* L.) [12], and sweet cherry (*Prunus avium* L.) [13].

Herbicide drift could be affected by many factors such as environmental conditions as well as the settings of spray machines [14,15,16,17]. Herbicide drift decreases the herbicide efficacy in area targeted for the application while negatively impacting the environment and agroecosystems due to nontargeted herbicide damage [18,19]. In susceptible crops, 2,4-D drift rates can induce synthase activity of 1 aminocyclopropane-1-carboxylic acid, which could increase ethylene formation [20]. Subsequently, increased ethylene content raises the level of abscisic acid that often triggers the process of aging in plants [21]. Various genotypes of crop species can behave differently to herbicide drift, mostly due to natural variation in tolerance ability. Therefore, it is important to study the effect of simulated drift rates of herbicides on different cultivars or genotypes of crops that are generally susceptible to non-target herbicide drift.

Over the past several years, a considerable number of studies have assessed the effect of auxin herbicides drift rates on different crops to understand the overall impact of drift rates on crop growth and yield production, as well as to identify relatively tolerant cultivars, genotypes or even wild accessions of some of the crops [22,23,24,25,26]. Recently, Zangoueinejad et al. (2019) evaluated the response of some selected wild tomato accessions to 2,4-D, dicamba, and quinclorac to the simulated drift rates of 11, 3, and 39 g ae ha^−1^, respectively. Both greenhouse and field experiments confirmed the tolerance of three wild tomato accessions (TOM199, TOM198, and TOM300) by approximately 85% to 90% to dicamba, while they were sensitive to 2,4-D and quinclorac [27]. Earlier, Hu et al. (2017) investigated the tolerance of several strawberry cultivars to different rates of terbacil (84, 168, and 336 g ae ha^–1^). Interestingly, all cultivars completely recovered from injury until 6 weeks after treatment (WAT) [28]. In another study, the tolerance of some tomato varieties to halosulfuron-methyl was assessed by exposition to the drift rates of 0, 34.7, and 70 g ae ha^−1^ [29]. At 6 WAT, of seven tomato varieties, E6203 displayed the most visual injury to halosulfuron-methyl at 70 g ae ha^−1^ in the first year of the experiment; however, all other varieties recovered from herbicide injury [29].

In this study, eighteen accessions and two commercial cultivars of melon commonly cultivated in Iran were screened for their tolerance or damage response to the application of the herbicide 2,4-D at three low rates simulating the typical drift rates. The objective was to determine the effects of simulated drift rates of 2,4-D on crop injury and yield in order to identify the tolerant melon accessions that can be recommended for cultivation in production systems susceptible to 2,4-D drift.

## 2. Materials and Methods

### 2.1. Experimental Setup, Treatments and Design

The experiment was conducted at the Liyan Kasht Agricultural Research and Development Center, Abtavil, Bushehr (29°05′ N 51°06′ E; elevation 29 m a. s. l) in partnership with University of Mohaghegh Ardabili, Ardabil, Iran, during the 2019 and 2020 growing seasons. The soil at the research station was silty clay loam with a pH of 7.4 and 1.4% organic matter. A randomized complete block experimental design with a factorial (two factors) arrangement was used, consisting of four replications. Factors included three 2,4-D rates and 20 melon genotypes, including eighteen wild accessions and two commercial cultivars (Table 1). The simulated herbicide drift rates included 1/10X (112.1 g ae ha^−1^), 1/100X (11.2 g ae ha^−1^), and 1/300X (3.7 g ae ha^−1^) of the full labeled rate (1X) of 2,4-D (1120.8 g ae ha^−1^) and also untreated plots used as control (modified from Culpepper et al. 2018).

Land was prepared by plowing and disking. Then, in both years of the experiment, the formation of 1-m-wide raised beds to a height of 20 cm was conducted, and subsequently, planting rows were covered using black plastic mulch (with a thickness of 25 µm). A basal fertilizer (NPK, 20:20:20; YaraRega™, Oslo, Norway) was incorporated into the farm soil at a rate of 150 kg ha^−1^. Overall, management practices including fertility, irrigation (using drip irrigation system by a water dropper interval of 20 cm), and standard pest control approaches were carried out modified from the southeastern US vegetable crop handbook to suit the local conditions [30]. Each plot measured 14 m × 12 m and consisted of four 14 m-long rows. The spacing between the center of row beds was 3 m. Additionally, to avoid the physical drift to adjacent plots, the distance between plots was 4 m and it was 6 m between the blocks. Melons were planted 70 cm apart resulting in 80 plants per plot.

Melons were seeded in 200-cell trays (size of each tray: 54 cm × 28 cm × 5 cm; the size of each cell: 2.4 cm × 2.4 cm/13cc) with potting mix (Mikskaar AS^®^ Company, Professional Substrate 300, Tallinn, Estonia) on 13 January 2019 and 15 January 2020 and grown in a greenhouse for approximately 5 wk. Melon seedlings were transplanted in the experimental field on 20 February 2019 and 22 February 2020. Application of 2,4-D (Weedar 64^®^; Nufarm, Inc., Chicago, IL, USA) at all drift rates was performed approximately 2 weeks after transplanting using a CO2-pressurized backpack sprayer equipped with flat spray nozzles (TeeJet 8002 XR; Spraying Systems, Wheaton, IL, USA), delivering 140 L ha^−1^ at 131 kPa. The wind speed at the time of herbicide treatment was 3 and 2 km h^−1^ in 2019 and 2020, respectively. Additionally, the air temperature at the time of herbicide application was 21–22 and 24–25 °C in 2019 and 2020, respectively. The relative humidity at the time of herbicide application was 65–70% in 2019 and 60–65% in 2020.

### 2.2. Observations

Visual crop injury symptoms, including stunting, epinasty, foliage deformations, and chlorosis, were determined and rated using a 0% to 100% scale, with 0% being no injury and 100% being total crop death [29]. Data were collected 3 and 6 weeks after treatment (WAT). To measure the yield production, melon fruits were manually harvested from two middle rows in each plot approximately 10 weeks after transplanting, when more than 85–90% of the fruit of different accessions and commercial cultivars were ripe, on 2 May 2019 and on 3 May 2020, and it was reported based on percent of control. It should be noted that as the yield potential of each accession was not the same, the percent of yield compared to control was measured using control of each accession and cultivar separately.

### 2.3. Statistical Analysis

Data were subjected to analysis of variance (ANOVA) using statistical program SAS version 9.4 (SAS Institute, Inc., Cary, NC, USA). Data were tested for normality using the PROC UNIVARIATE procedure and natural logarithmic transformations were performed as necessary. Levene’s test was used to verify the homogeneity of the variances. Replications and years of the experiment were considered as the random effects, and the herbicide application rates and wild melon accessions were considered as the fixed effects. Subsequently, data for crop injury and melon yield production were reported by year, since the random effect of year and its interaction with herbicide treatment, was significant for these parameters. Fisher’s protected least significant difference (LSD) test was used to separate means at the 5% level of probability.

## 3. Results and Discussion

**Figure 1:**
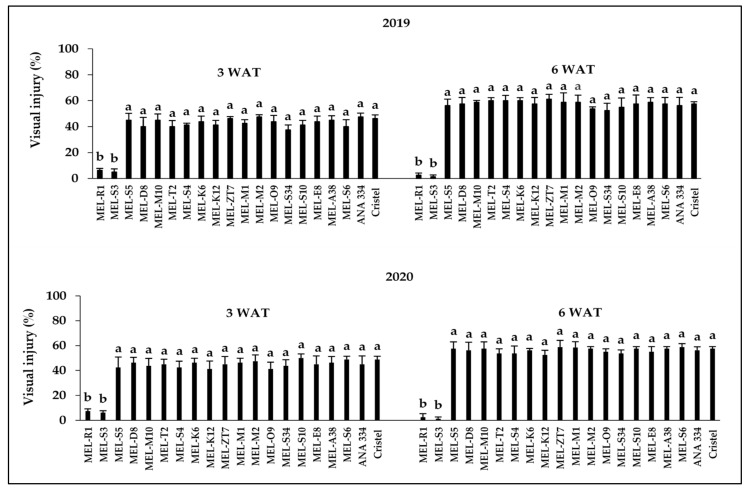
Response of melon accessions and commercial cultivars to 2,4-D application at the rate of 3.7 g ae ha^−^^1^ 3 and 6 weeks after treatment during 2019 and 2020. Means in each chart followed by different letters are significantly different at *p* ≤ 0.05 using LSD test.

**Figure 2:**
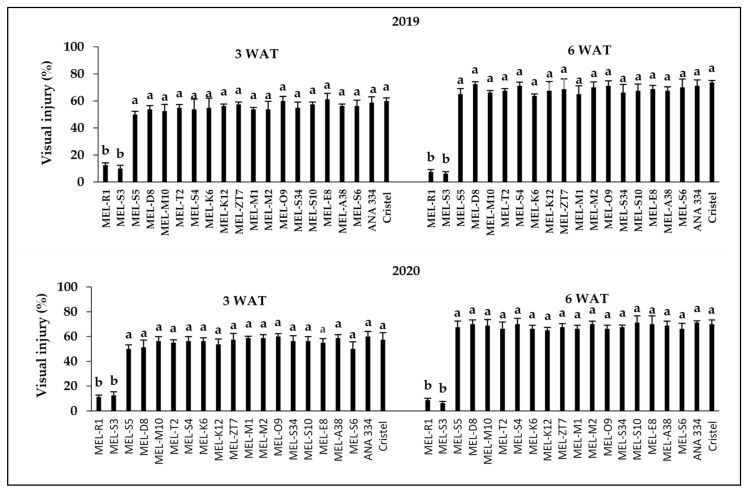
Response of melon accessions and commercial cultivars to 2,4-D application at the rate of 11.2 g ae ha^−^^1^ 3 and 6 weeks after treatment during 2019 and 2020. Means in each chart followed by different letters are significantly different at *p* ≤ 0.05 using LSD test.

**Figure 3:**
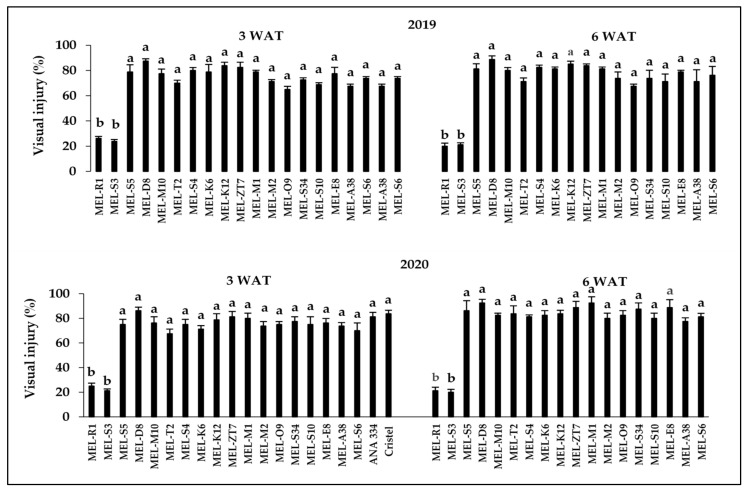
Response of melon accessions and commercial cultivars to 2,4-D application at the rate of 112.1 g ae ha^−^^1^ 3 and 6 weeks after treatment during 2019 and 2020. Means in each chart followed by different letters are significantly different at *p* ≤ 0.05 using LSD test.

The interaction between herbicide rate and melon accessions was significant (*p* < 0.001) at 3 and 6 WAT in both years of the experiment. The level of visual injury increased with increasing rate of 2,4-D (Figure 1, Figure 2 and Figure 3). After the application of 2,4-D at all three rates, epinasty of stems and leaves, as well as necrosis following chlorosis in the youngest leaves, were the most explicit visual symptoms in damaged accessions (data not shown). Those symptoms are typical of the auxinic herbicides injury [13,26,31]. Epinasty of eggplant’s (*Solanum melongena* L.) youngest leaves and even fruits after the application of aminocyclopyrachlor increased by increasing the simulated drift rates from 0.0001 to 10 g ae ha^−^^1^ [2]. Leaf curling and stem epinasty were observed as the tomato’s responses to simulated drift rates (1.68–13.44 g ae ha^−^^1^) of 2,4-D [22]. Therefore, the response to the simulated rates used in our study was typical of similar rates used in previous studies.

### 3.1. Effect on Crop Injury

Of all 18 melon accessions, MEL-S3 and MEL-R1 were the most tolerant accessions to 2,4-D at all three drift rates. After the application of 2,4-D at the rate of 3.73 g ae ha-1, these accessions showed only almost 6% and 2% injury at 3 and 6 WAT, respectively, in both years of the experiment (Figure 1). The recorded injury for these increased up to almost 11% and 7% to 2,4-D at the rate of 11.2 g ae ha^−1^ at 3 and 6 WAT, respectively (Figure 2). By increasing the herbicide drift rate up to 112.1 g ae ha^−1^, the level of visual injury for both accessions increased by approximately 25% and 20% at 3 and 6 WAT, respectively, in 2019 and 2020 (Figure 3). However, the scenario was different regarding the most susceptible accessions after application of each herbicide drift rate. The most sensitive accession was MEL-M2 to the 2,4-D treatment at the drift rate of 3.7 g ae ha^−1^ at 3 WAT, while MEL-ZT7 was the most susceptible accession, with 61% recorded visual injury, at 6 WAT in 2019 (Figure 1). In 2020, MEL-S6 displayed the highest level of injury by approximately 50% to 2,4-D at the stimulated drift rate of 3.7 g ae ha-1 3 WAT (Figure 1). MEL-ZT7 and MEL-S6 were the most sensitive accessions, with approximately 60% recorded injury, at 6 WAT in 2020 (Figure 1).

There was some variation in genotypic sensitivity to drift rates across different assessment stages and years, but the overall trend did not differ much. In 2019, MEL-E8 appeared to be the most sensitive accession with about 60% injury at 3 WAT at 11.2 g ae ha^−1^ of 2,4-D, while at 6 WAT, MEL-D8 showed the highest injury response (72%) at the same drift rate (Figure 2). After the 2,4-D treatment at the rate of 11.2 g ae ha^−1^, MEL-O9 showed 60% injury, which was the most sensitive accession at 3 WAT, while 71% visual injury was recorded for MEL-S10 at 6 WAT, as the most sensitive accessions, in 2020 (Figure 2). Moreover, MEL-K12 was the most sensitive accession to the 2,4-D drift rate of 112.1 g ae ha^−1^ at 3 WAT in 2019 (Figure 3). At 6 WAT, approximately 90% visual injury was recorded for MEL-D8, which indicated the most susceptibility, in 2019 (Figure 3). MEL-S3 represented the highest injury level by 86% at 3 WAT in 2020 (Figure 3). The highest visual injury level was recorded for MEL-D8 and MEL-M1 by 92%, the most sensitive accessions, at 6 WAT in 2020 (Figure 3).

The observed visual injuries for both commercial cultivars, ANA 334 and Cristel, in response to herbicide treatment at all three doses were ranked among the highest damage levels recorded by accessions, therefore, these cultivars were susceptible to all 2,4-D drift rates (Figure 1, Figure 2 and Figure 3). In line with our findings, Hand et al. [32] reported that by increasing the application rate of 2,4-D or dicamba from 1/25X to 1/7X their recommended field use rates (1120 and 560 g ae ha^−1^, respectively), the level of visual injury in cantaloupe (*Cucumis melo* L.) increased up to 67%, 110%, and 100% at 18, 31, and 54 days before harvesting, respectively.

In another study, visual injury increased significantly by increasing the 2,4-D rate from 1/200X to 1/2X the manufacturer’s recommended use rate (1090 g ae ha^−1^) at 28 and 56 days after treatment in some ornamental, fruit, and nut plant species [26]. In a separate study, broccoli (*Brassica oleracea* L.) and bell pepper (*Capsicum annuum* L.) recorded higher injury level when the rate of 2,4-D treatment increased from 2.1 to 16.8 g ae ha^−1^ [33].

In the present study, of the 18 melon accessions, MEL-S3 and MEL-R1 exhibited lower susceptibility to 2,4-D than others, with a recorded less than 30% injury at 3 and 6 weeks after the application of 2,4-D at all three rates of 112.1, 11.2, and 3.7 g ae ha^−1^ in 2019 and 2020. Importantly, the results reflected the potential of both these tolerant accessions to recover from injury over time, while visual injury of all other accessions increased gradually from 3 WAT to 6 WAT in both years of the experiment after the application of herbicide at all three rates (Figure 1, Figure 2 and Figure 3).

Variation in tolerance levels across different accessions might be due to genetic tolerance ability as well as the environmental interactions. Recently, Zangoueinejad et al. [34] demonstrated that expression levels of gene TIR1 between dicamba-tolerant tomato wild accessions and susceptible cultivars differed significantly, but no difference was recorded in the expression levels of the genes AFB1 and AFB2. Wild tomato accessions displayed lower herbicide absorption than commercial cultivars after the application of dicamba at drift rate of 2.8 g ae ha^−1^ [35]. It was reported leaf characteristics such as leaf length/width ratio and trichome density potentially influenced herbicide absorption [35]. Another study found that the 2,4-D-resistant corn poppy (*Papaver rhoeas* L.) biotypes translocated less [^14^C]-2,4-D compared to sensitive plants [36]. Additionally, the 2,4-D-resistant corn poppy showed lower ethylene production than in susceptible plants after treatment with 2,4-D [36]. Figueiredo et al. [37] reported that the cytochrome P450-mediated rapid 2,4-D metabolism is an influencing element to resistance in a common waterhemp (*Amaranthus tuberculatus* L.) population.

### 3.2. Effect on Melon Fruit Yield

**Figure 4:**
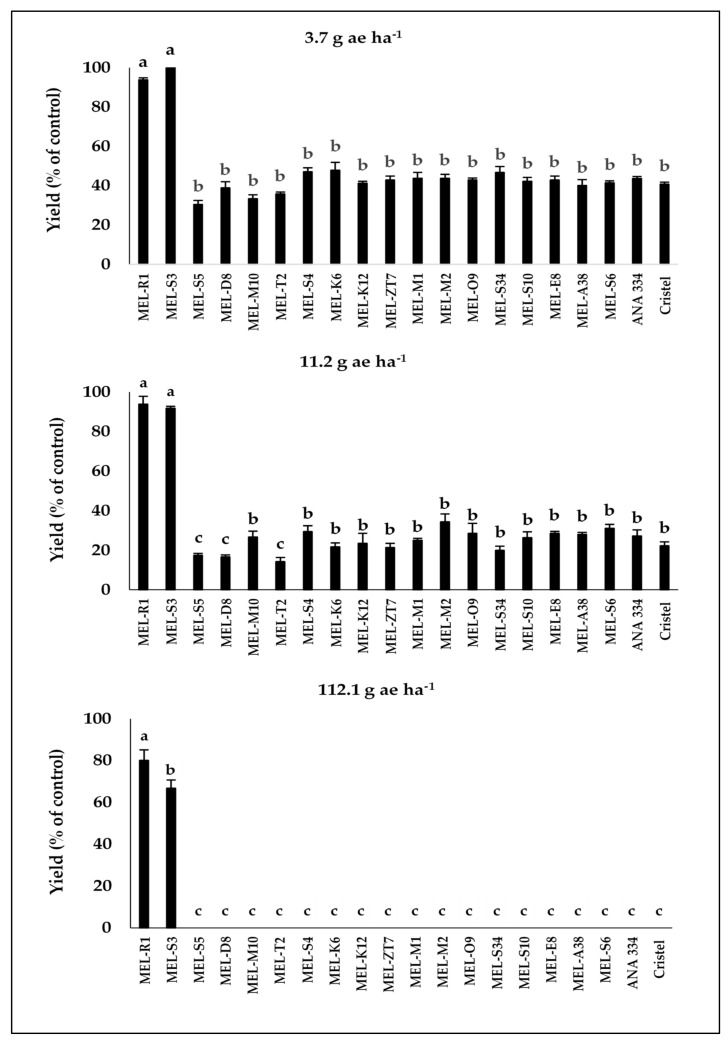
The fruit yield production compared to untreated control in melon accessions and commercial cultivars in response to different drift rates of 2,4-D during 2019. Means in each chart followed by different letters are significantly different at *p* ≤ 0.05 using LSD test.

**Figure 5:**
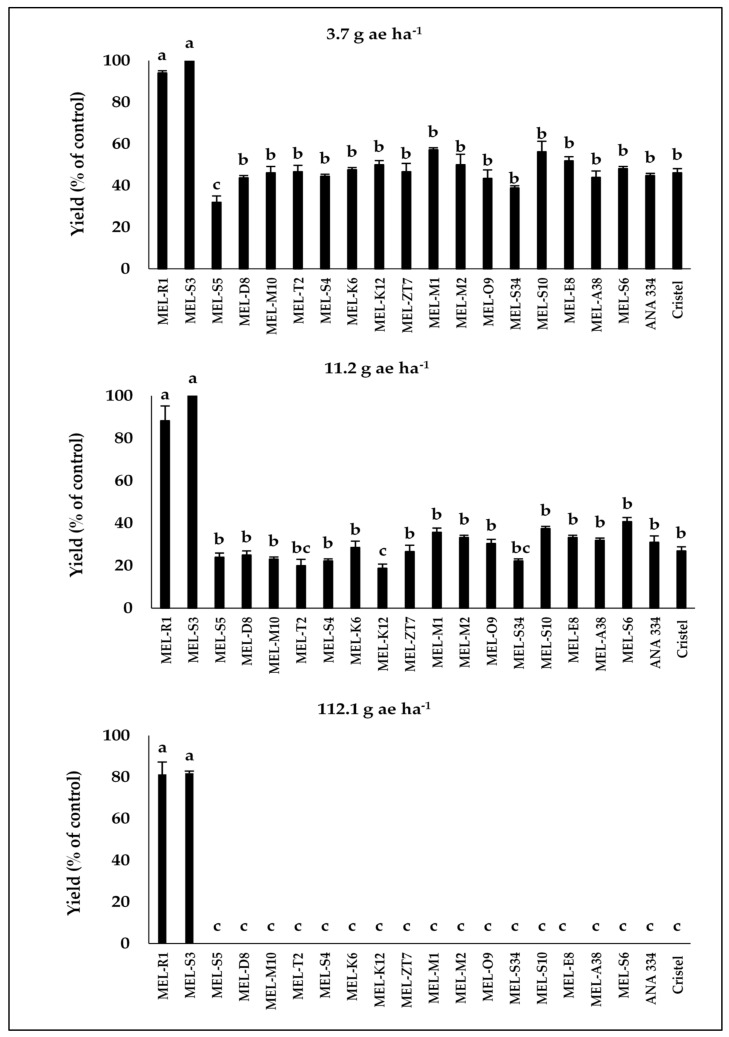
The fruit yield production compared to untreated control in melon accessions and commercial cultivars in response to different drift rates of 2,4-D during 2020. Means in each chart followed by different letters are significantly different at *p* ≤ 0.05 using LSD test.

As expected, based on the visual injury results, only MEL-R1 and MEL-S3, the most tolerant accessions, displayed fruit yield production at 80% and 67% of their control levels, respectively, in 2019 and also 81% and 82% of their control levels, respectively, in 2020 after treatment by 2,4-D at the rate of 112.1 g ae ha^−1^ (Figure 4 and Figure 5).

More importantly, MEL-S3 and control plots did not show different fruit yield production after 2,4-D treatment at the rate of 112.1 g ae ha^−1^ in 2020 (Figure 4 and Figure 5). Generally, after the application of 2,4-D at the rates of 11.2 and 3.7 g ae ha^−1^, all accessions and commercial cultivars showed yield production in 2019 and 2020, while the yield production of each melon accession as well as commercial cultivars was significantly lower than their control plots, except for MEL-R1 and MEL-S3 (Figure 4 and Figure 5).

Previously, after the application of 2,4-D at the rate of 2.1 g ae ha^−1^, root crops such as carrot (*Daucus carota* L.), radish (*Raphanus sativus* L.), rutabaga (*Brassica napus* L.), and turnip (*Brassica rapa* L.), and also tomato, varied as the most susceptible crops, presenting significant herbicide damage in roots or fruits [18]. The root crops and also tomato fruit production was not significantly different compared to untreated control plots after treatment with 2,4-D at the drift rate of 20.8 g ae ha^−1^ [18]. Noticeably, after the application of 2,4-D at the drift rate of 208 g ae ha^−1^, all crops significantly showed lower yield production than their untreated control plots [18]. By increasing the rate of 2,4-D from 44.8 to 160 g ae ha^−1^, the cantaloupe fruit number and fruit weight reduced by 32% and 50%, respectively [32].

Although it is important to mitigate or minimize the negative effects of herbicide drift, avoiding it happening in the first place is extremely important. For instance, optimizing all spray settings along with reducing the negative effects of environmental conditions on the spray process via performing it at the optimum time could significantly reduce the occurrence of herbicide drift. On the other hand, research into mitigation strategies should also be prioritized. The use of herbicide-tolerant crops within well-defined agroecological zones can improve weed management and preserve crop yield potential [27,28,29,38,39]. Good stewardship for herbicide application and herbicide-tolerant crops is important for sustainable weed management.

## 4. Conclusions

In conclusion, two melon accessions, including MEL-R1 and MEL-S3 showed the lowest level of visual injury out of all accessions. More importantly, those accessions recovered from injury after 2,4-D treatment at all three simulated drift rates over time. On the other hand, both accessions demonstrated a lower level of visual injury (<30%) even when 2,4-D was applied at the highest rate of 112.1 g ae ha^−1^. Therefore, evidence affirmed that these accessions could be considered as tolerant lines to simulated drift rates of 2,4-D. These can be recommended broadly for commercial cultivation while also considered in breeding programs to develop cultivars with high 2,4-D tolerance, suited for cultivation in drift-prone areas.

## Figures and Tables

**Table 1:** Information about the origin and source of Iranian melon accessions and two commercial cultivars.

Accession Code	Origin	Seed Source
MEL-S10	Bushehr	Local farmer
MEL-E8	Bushehr	Local farmer
MEL-S6	Garmsar	Local farmer
MEL-A38	Garmsar	Local store
MEL-S3	Esfahan	Local store
MEL-S5	Esfahan	Local store
MEL-D8	Esfahan	Local store
MEL-M10	Neyshabur	Local farmer
MEL-K6	Mashhad	Local store
MEL-T2	Mashhad	Local farmer
MEL-K12	Torbet Jam	Local store
MEL-M1	Torbet Jam	Local farmer
MEL-O9	Torbet Jam	Local store
MEL-S4	Saveh	Local farmer
MEL-ZT7	Shabestar	Local farmer
MEL-M2	Shabestar	Local farmer
MEL-S34	Zanjan	Local store
MEL-R1	Kashan	Local farmer
**Cultivar**	**Origin**	**Seed source**
ANA 334	Italy	Local store
Cristel	France	Local store

## Data Availability

The data presented in this study are available on request from the corresponding author.
